# Association of achieved LDL-C levels with global coagulability and clinical outcomes in patients with acute coronary syndrome receiving PCSK9 inhibitors: a prospective cohort study

**DOI:** 10.3389/fcvm.2026.1804585

**Published:** 2026-06-10

**Authors:** Tianfu Fu, Song Yao, Shaojiang Zhang, Hui Linghu

**Affiliations:** 1 Department of Emergency, The First People's Hospital of Zunyi City (The Third Affiliated Hospital of Zunyi Medical University), Zunyi City, Guizhou Province, China; 2 Department of Cardiology, The First People's Hospital of Zunyi City (The Third Affiliated Hospital of Zunyi Medical University), Zunyi City, Guizhou Province, China

**Keywords:** acute coronary syndrome, bleeding risk, coagulability, low LDL-C, PCSK9 inhibitors, platelet reactivity

## Abstract

**Background:**

The safety of achieving ultra-low low-density lipoprotein cholesterol (LDL-C) levels in acute coronary syndrome (ACS) patients on dual antiplatelet therapy (DAPT), particularly regarding global coagulability, specific platelet reactivity, and bleeding risk, requires further validation.

**Methods:**

In this prospective, open-label, single-center cohort study, 120 post-PCI ACS patients treated with PCSK9 inhibitors and DAPT were enrolled. After a 2-week run-in, patients were stratified by their achieved LDL-C levels into a Low LDL-C group (≤0.78 mmol/L) and a Non-Low LDL-C group (>0.78 mmol/L). Global coagulability and specific platelet reactivity were serially assessed via thromboelastography and rapid platelet function assays over 12 months. The primary safety endpoint was clinically relevant bleeding [Bleeding Academic Research Consortium (BARC) types 2, 3, or 5].

**Results:**

The incidence of clinically relevant bleeding was comparable between the Low LDL-C (6.7%) and Non-Low LDL-C (5.0%) groups (risk difference 1.7%, 95% CI: −5.8% to 9.2%). Due to the sparse number of overall bleeding events (*n* = 7), the pre-specified non-inferiority analysis was severely underpowered; therefore, these safety findings are reported descriptively. Exploratory time-to-event analysis showed no significant difference in overall bleeding risk (log-rank *P* = 0.72). Global coagulability and specific platelet reactivity parameters remained stable throughout follow-up, with no significant intergroup differences (all *P* > 0.05). Major adverse cardiovascular events (MACE) occurred in 6.7% of the Low LDL-C group and 13.3% of the Non-Low LDL-C group (HR 0.48, 95% CI: 0.15–1.55; log-rank *P* = 0.22).

**Conclusion:**

In ACS patients receiving PCSK9 inhibitors and DAPT, achieving an LDL-C level ≤0.78 mmol/L was associated with neither an increased bleeding risk nor significant alterations in global coagulability and specific platelet reactivity. These exploratory observational findings descriptively support the clinical safety of profound lipid-lowering in this high-risk population.

## 1 Introduction

Acute coronary syndrome (ACS), a severe clinical presentation of coronary artery disease, remains a major contributor to global cardiovascular mortality, underscoring the need for comprehensive management strategies to optimize outcomes (1, 2). Percutaneous coronary intervention (PCI) serves as the foundational treatment for achieving prompt coronary revascularization. It is important to note, however, that the PCI procedure can itself trigger platelet activation and enhance aggregation, thereby elevating the risk of subsequent thrombotic events (3). Consequently, optimal post-PCI care integrating potent antiplatelet therapy with intensive lipid-lowering strategies, is crucial for improving long-term prognosis.

Elevated low-density lipoprotein cholesterol (LDL-C) is a well-established, causative driver of atherosclerotic cardiovascular disease (4). Beyond its role in plaque formation, evidence suggests a direct interplay between lipids and thrombosis. Oxidized LDL components can promote platelet activation, and hypercholesterolemia is associated with enhanced platelet reactivity (5, 6). Conversely, lipid-lowering therapy, particularly with statins, has been shown to normalize platelet function, underscoring the mechanistic link between LDL-C levels and hemostasis (7). Mechanistically, this link is heavily dependent on the P2Y12 receptor pathway. Systemic LDL-C directly modulates platelet membrane cholesterol content, which preferentially aggregates into lipid rafts. These microdomains are strictly required for the optimal downstream G*α*i signaling of the ADP-activated P2Y12 receptor (8). Preclinical evidence demonstrates that hypercholesterolemia enriches these lipid rafts, thereby amplifying P2Y12-mediated platelet hyperreactivity and thrombosis (9). Conversely, profound lipid-lowering theoretically depletes membrane cholesterol and disrupts lipid raft integrity, which could dampen P2Y12 signaling. This mechanistic crosstalk builds the core clinical hypothesis of our study: in post-PCI ACS patients already subjected to intense pharmacological P2Y12 blockade (DAPT), the drastic LDL-C reduction achieved by PCSK9 inhibitors might synergistically impair platelet reactivity, thereby inheriting a heightened risk for clinically relevant bleeding.

Proprotein convertase subtilisin/kexin type 9 (PCSK9) is a key regulator of LDL cholesterol (LDL-C) metabolism. It elevates circulating LDL-C levels by promoting the degradation of hepatic LDL receptors. Monoclonal antibody inhibitors of PCSK9 (PCSK9i), including evolocumab and alirocumab, potently reduce LDL-C by 50%–70% and have proven cardiovascular benefits in major outcome trials (10, 11). Beyond lipid metabolism, PCSK9 appears to intersect with platelet biology. Preclinical evidence suggests that PCSK9 can bind directly to platelet CD36, enhancing activation via NADPH oxidase-related pathways, an effect amplified by LDL-C (12, 13). Clinically, elevated plasma PCSK9 levels correlate with heightened platelet reactivity and worse cardiovascular outcomes in ACS patients, indicating a potential pro-thrombotic role (14).

Current guidelines endorse intensive LDL-C lowering for very high-risk patients, advocating targets below 1.4 mmol/L and a > 50% reduction from baseline (15). However, the safety of achieving low LDL-C, particularly regarding bleeding, remains under scrutiny. An observational study by Yang et al. found that in ACS patients on intensive antithrombotic therapy, in-hospital LDL-C levels <1.8 mmol/L were associated with an increased risk of major bleeding (16). This raises a pivotal, unanswered question: could achieving profound LDL-C reduction via PCSK9i carry a similar risk?

While landmark trials (FOURIER, ODYSSEY OUTCOMES) established the efficacy and overall safety of PCSK9i (10, 11, 17), they were not designed to assess coagulability and platelet reactivity or the specific risks of attaining LDL-C levels well below current targets. Furthermore, these trials included limited East Asian participants, a population that may exhibit distinct pharmacodynamic responses. Thus, the impact of sustained, low LDL-C levels on coagulability, platelet reactivity and clinical bleeding risk in contemporary ACS patients treated with PCSK9i and dual antiplatelet therapy (DAPT) is still uncertain.

To address this gap in evidence, we designed a prospective cohort study. We hypothesized that for post-PCI ACS patients, achieving a low LDL-C level via PCSK9i therapy would not negatively impact coagulability and platelet reactivity or increase the incidence of clinically relevant bleeding events compared to maintaining a moderately low level. This study aimed to evaluate the influence of intensively lowering LDL-C via PCSK9 inhibitors on global coagulability using thromboelastography (TEG) and to determine the corresponding clinical safety over a 12-month period.

## 2 Methods

### 2.1 Study design

This was a single-center, open-label, prospective cohort study designed to assess the impact of PCSK9 inhibitors on coagulability, platelet reactivity and safety profiles in ACS patients attaining low LDL-C levels. Approved by the Institutional Review Board of The First People's Hospital of Zunyi City, the study was conducted in compliance with the ethical principles of the 2013 revision of the Declaration of Helsinki. Written informed consent was obtained from all participants before any study procedures were initiated.

### 2.2 Study population

Between December 2023 and January 2025, consecutive patients aged 18–75 years, diagnosed with ACS per universal definition, were evaluated for enrollment. The explicit primary inclusion criterion was the successful completion of percutaneous coronary intervention (PCI) for at least one culprit coronary lesion, ensuring all participants shared a standardized baseline requirement for intensive DAPT. Conversely, patients with ACS managed solely with medical therapy without revascularization, or those undergoing coronary artery bypass grafting (CABG), were strictly excluded to maintain profound cohort homogeneity; conservative medical management lacks acute stent-induced thrombogenicity, whereas CABG introduces severe surgical and cardiopulmonary bypass-related coagulopathies that would critically confound the standardized assessment of global coagulability and specific platelet reactivity. Upon enrollment, all patients were required to be on guideline-directed DAPT (aspirin combined with a P2Y12 inhibitor) and a stable moderate-intensity statin regimen (e.g., atorvastatin ≤20 mg/day or equivalent), or have documented severe statin intolerance, in combination with a PCSK9 inhibitor (evolocumab or alirocumab) (18, 19). Moderate-intensity statin therapy was strictly selected over high-intensity regimens because East Asian populations exhibit greater systemic exposure and a significantly higher risk of statin-associated muscle symptoms and hepatotoxicity at high doses compared to Western cohorts (20). Thus, moderate-intensity statins combined with a PCSK9 inhibitor represent the standard strategy for safely achieving intensive lipid-lowering targets, as explicitly recommended by the contemporary 2023 Chinese Guidelines for Lipid Management (21). Consequently, for the approximately 8% of the cohort presenting with documented severe statin intolerance, PCSK9 inhibitor therapy without concomitant statins was prescribed as the guideline-directed alternative for optimal lipid management. Specifically, ticagrelor was chosen as the predominant potent P2Y12 inhibitor since prasugrel is not widely available in our regional routine clinical practice. Clopidogrel was specifically prescribed for patients considered to have a high bleeding risk (HBR), a history of stroke/transient ischemic attack, or those intolerant to potent P2Y12 inhibitors, in strict accordance with contemporary guidelines (18, 22).

Key exclusion criteria were: (1) ACS managed solely with medical therapy without revascularization, or those undergoing CABG (rationale detailed above); (2) recent or active bleeding; (3) known hypersensitivity/contraindication to study drugs; (4) concomitant use of other non-statin lipid-lowering therapies; (5) pre-existing hematologic disorders (e.g., thrombocytopenia); (6) anticipated need for long-term oral anticoagulation; (7) concurrent participation in another interventional clinical trial; (8) any condition potentially compromising protocol compliance (e.g., severe psychiatric disorder); or (9) pregnancy.

Withdrawal was defined by consent withdrawal, loss to follow-up, severe adverse events leading to treatment discontinuation, or protocol non-compliance. All analyses will follow the intention-to-treat (ITT) principle, analyzing participants according to their original group assignment regardless of subsequent adherence.

### 2.3 Group allocation

As a strictly observational cohort, the clinical decision to initiate PCSK9 inhibitor therapy was made entirely by the treating physicians in accordance with contemporary lipid management guidelines for very high-risk ACS patients, independent of study participation. After this clinical decision and the initial baseline coagulability and platelet reactivity assessment, patients were enrolled and longitudinally followed. The specific drugs administered as part of their routine clinical care were either evolocumab (Repatha®; 140 mg in 1 mL; Amgen Inc.) or alirocumab (Praluent®; 75 mg in 1 mL; Sanofi). In accordance with current clinical practice guidelines and evidence from pivotal trials, the standard dosing regimen was administered: 140 mg of evolocumab or 75 mg of alirocumab given via subcutaneous injection every two weeks (18).

Patients were stratified into two groups based on the achieved LDL-C level after a 2-week run-in period: a Low group (LDL-C ≤ 0.78 mmol/L) and a Non-Low group (LDL-C > 0.78 mmol/L). To ensure optimal and equal statistical power for both cohorts, a stratified quota-based sampling strategy was prospectively employed, targeting exactly 60 patients per group. Because of the profound lipid-lowering efficacy of PCSK9 inhibitors, the 60-patient quota for the low group was fulfilled relatively early. Consequently, screening was extended specifically to recruit sufficient patients who remained >0.78 mmol/L despite therapy, until the Non-Low quota was also strictly met. This threshold of 0.78 mmol/L (equivalent to 30 mg/dL) was selected based on substantial evidence from landmark cardiovascular outcome trials. It was established as a key prespecified analytical cutoff in the IMPROVE-IT trial, which validated the safety and efficacy of achieving such Low LDL-C concentrations in high-risk patients (23). The clinical relevance of this threshold was further solidified in the era of PCSK9 inhibitors by the FOURIER trial, in which 0.78 mmol/L represented the median LDL-C level achieved in patients treated with evolocumab (10). Therefore, this value provides a well-documented, evidence-based cutoff for investigating the effects of intensive lipid-lowering therapy on global coagulability, specific platelet reactivity, and clinical safety in a contemporary ACS cohort.

### 2.4 Data collection and laboratory measurements

Comprehensive baseline data were meticulously collected upon patient enrollment, encompassing demographics, medical history, cardiovascular risk factors, and medications on admission. Follow-up assessments were conducted at scheduled intervals: on the day of PCSK9i initiation (baseline), and at 1, 3, 6, and 12 months thereafter. All data were recorded on standardized case report forms (CRFs) by trained study coordinators. To ensure data quality, regular monitoring was performed to check for completeness, consistency, and accuracy, with discrepancies resolved by cross-referencing with source documents.

At each follow-up visit, venous blood samples were drawn under standardized conditions. Routine laboratory tests included a complete blood count (CBC) with platelet enumeration, a coagulation profile, and serum biochemistry, all processed at the central laboratory of our institution. In addition, specific assays for coagulability and platelet reactivity were performed, including platelet count, clot retraction test (CRT), whole blood coagulation analysis (WBCA), activated clotting time (ACT), platelet count ratio (PCR), and rapid platelet function assay (RPFA).

For a detailed evaluation of coagulation dynamics, TEG was performed using the TEG® 5,000 Hemostasis Analyzer System (Haemonetics Corporation, Braintree, MA, USA). The analysis focused on key parameters reflecting the entire coagulation cascade: R-time (reaction time), K-time (coagulation time), MA (maximum amplitude), angle (*α*), and G-value. All TEG procedures followed the manufacturer's instructions, with regular quality control checks using standardized reagents to ensure the reliability and consistency of the results.

Data pertaining to all pre-specified clinical endpoints and safety events were systematically collected during follow-up. This was accomplished through direct patient interviews during clinic visits, structured telephone calls, and a thorough review of the patients' electronic medical records (EMR) and any relevant hospital discharge summaries. All potential endpoint events were recorded, including MACE, defined as the composite of all-cause mortality, non-fatal myocardial infarction, or non-fatal stroke, as well as bleeding events (assessed using the BARC scale). Any adverse events (AEs) or serious adverse events (SAEs) reported by the patient or identified by the investigators were documented in detail, irrespective of their perceived relationship to the study treatment.

Data quality was maintained through several mechanisms. All research personnel were trained on the study protocol and standardized data collection procedures. Data were entered into a centralized, password-protected electronic Case Report Form (eCRF) system. The system had built-in checks for data range and consistency. A designated clinical research monitor periodically reviewed the collected data for accuracy and completeness against source documents to ensure adherence to the protocol.

### 2.5 Study endpoints

The primary endpoint of this study was the incidence of clinically relevant bleeding events within one year after group allocation, evaluated in a non-inferiority framework. Clinically relevant bleeding was strictly defined according to the Bleeding Academic Research Consortium (BARC) classification as the composite of BARC type 2, 3, or 5 bleeding (24). BARC type 2 is defined as any overt, actionable bleeding not meeting the criteria for type 3 or 5; type 3 involves significant hemoglobin drop, need for transfusion, or hemodynamic compromise; and type 5 is fatal bleeding.

Secondary endpoints were categorized into two major areas. The first concerned the dynamic evolution of global coagulability and specific platelet reactivity over the one-year follow-up. Serial TEG measurements served as the primary assessment tool for evaluating overall coagulation dynamics and clot strength, while the rapid platelet function assay (RPFA, VerifyNow P2Y12) was specifically utilized to assess platelet reactivity in the context of DAPT. The second was a composite MACE endpoint (all-cause mortality, non-fatal MI, or non-fatal stroke), defined and adjudicated in accordance with contemporary ESC guidelines (18).

Pre-specified safety endpoints facilitated a thorough safety profile. These involved severe bleeding, evaluated using multiple standardized criteria: BARC (types 3, 5) (24), TIMI (major/minor) (25), GUSTO (moderate/severe/life-threatening) (26), and ISTH (major) (27). Further ischemic endpoints, analyzed on a time-to-first-event basis, included a composite of cardiovascular death, MI, ischemic stroke, and definite/probable stent thrombosis (ARC definitions) (28).

### 2.6 Sample size

A prospective enrollment of 120 eligible patients is planned. The calculation was anchored on the primary non-inferiority safety endpoint (one-year incidence of BARC 2/3/5 bleeding). Based on an assumed 5.0% event rate in the control arm (22), a non-inferiority margin of 4.5%, a one-sided alpha of 0.025, and 80% power, at least 51 patients per group were needed. Accounting for an anticipated 15% dropout rate, the final target was set at 60 patients per group (*N* = 120 total).

### 2.7 Statistical analysis

Data analysis will utilize SPSS software (v26.0, IBM Corp.). Continuous variables will be described as mean ± SD (normal distribution) or median (IQR) (non-normal distribution), with normality evaluated via the Shapiro–Wilk test. Categorical variables will be shown as n (%). Group comparisons for baseline characteristics will employ independent-sample t-tests/Mann–Whitney U tests for continuous variables and chi-square/Fisher's exact tests for categorical variables, based on data distribution.

For the primary endpoint (clinically relevant bleeding, BARC types 2, 3, or 5), a non-inferiority analysis will be performed. The risk difference between groups and its 95% confidence interval will be calculated and compared against a pre-defined non-inferiority margin. Time-to-event data for both primary and secondary endpoints will be evaluated using Kaplan–Meier survival curves, with comparisons made via the log-rank test. Cox proportional hazards models will be used to estimate hazard ratios (HRs) with 95% confidence intervals, adjusting for relevant clinical covariates.

Longitudinal changes in coagulability and platelet reactivity parameters and LDL-C levels across follow-up visits will be analyzed using repeated-measures analysis of variance (ANOVA) or linear mixed-effects models, with assessment of group-by-time interaction. Correlation analyses (Pearson or Spearman) will be used to explore relationships between the magnitude of LDL-C reduction and changes in coagulability and platelet reactivity. Subgroup analyses will be performed based on key baseline characteristics. To further address potential confounding inherent in our non-randomized group stratification, multivariable Cox proportional hazards regression was performed for the primary bleeding endpoint, adjusting for clinically relevant covariates including age, sex, P2Y12 inhibitor type (ticagrelor vs. clopidogrel), and proton pump inhibitor use. Furthermore, as a sensitivity approach, an inverse probability of treatment weighting (IPTW) analysis was conducted based on propensity scores derived from baseline clinical, pharmacological, and procedural characteristics. A two-sided *p*-value < 0.05 will be considered statistically significant for superiority analyses. All analyses will follow the ITT principle.

## 3 Results

### 3.1 Study population and baseline characteristics

**Figure 1:**
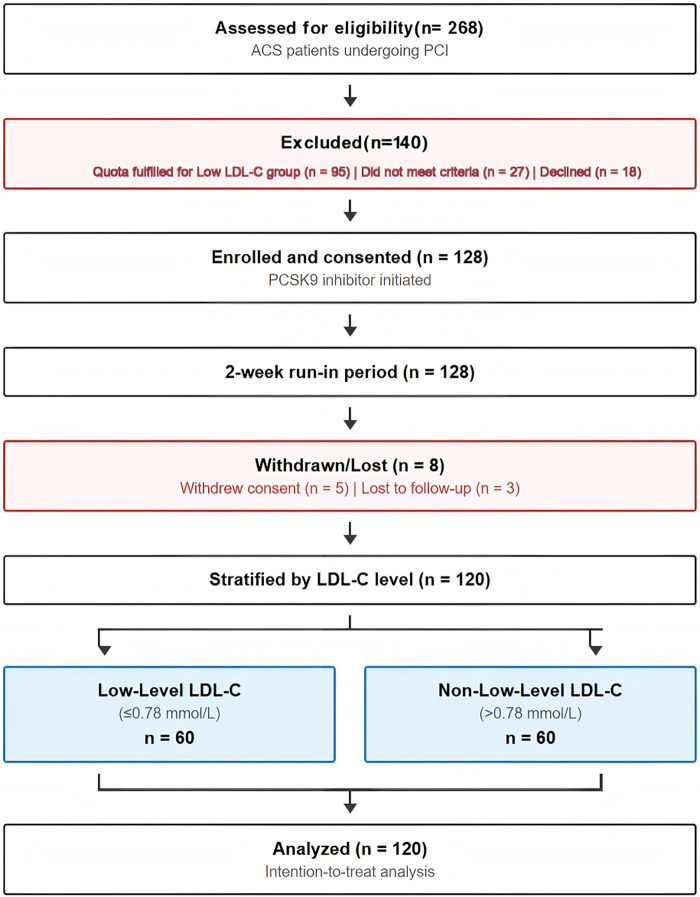
Flow diagram of patient enrollment and group allocation.

Between December 2023 and January 2025, a total of 268 ACS patients post-PCI were screened. Due to the stratified quota-based enrollment design, once the Low LDL-C group (≤0.78 mmol/L) reached its pre-specified target of 60 patients, 95 subsequent eligible patients who successfully achieved this low target were safely managed but excluded from this specific comparative cohort. After excluding another 45 patients (27 who did not meet the inclusion criteria and 18 who declined participation), an initial cohort of 128 patients provided informed consent and were enrolled. During the initial 2-week run-in period after starting PCSK9 inhibitor therapy, 8 patients withdrew consent or were lost to follow-up. The remaining 120 patients completed the run-in phase and successfully matched our target enrollment: 60 patients in the Low LDL-C group and 60 patients in the Non-Low LDL-C group. All 120 patients were included in the intention-to-treat analysis. The study flow is illustrated in Figure 1.

Baseline demographic, clinical, laboratory, and medication profiles are shown in Table 1. The two groups were well-balanced across all measured parameters (all *P* > 0.05). The overall cohort had a mean age of 62.8 ± 8.0 years, and 77.5% were male. The distribution of cardiovascular risk factors, such as hypertension, diabetes mellitus, and smoking history, was similar between groups. Laboratory values at admission, including mean LDL-C levels prior to PCSK9 inhibitor initiation, were comparable. Furthermore, no significant differences were observed in the prescription of guideline-recommended medications at enrollment, including DAPT, statins, beta-blockers, and ACEIs/ARBs (all *P* > 0.05).

**Table 1:** Baseline characteristics.

Characteristic	Low LDL-C roup *n* = 60)	Non-Low L D-C Grop (*n* = 60)	Statistic^a^	*P*-value
Demographics				
Age, years	62.5 ± 8.1	63.1 ± 7.9	t = −0.41	0.68
Male sex, *n* (%)	45 (75.0)	48 (80.0)	*χ*^2^ = 0.43	0.51
Body Mass Index, kg/m^2^	26.8 ± 3.5	27.1 ± 3.2	t = −0.49	0.62
Clinical History & Risk Factors				
Hypertension, *n* (%)	40 (66.7)	42 (70.0)	χ^2^ = 0.15	0.70
Diabetes Mellitus, *n* (%)	18 (30.0)	20 (33.3)	χ^2^ = 0.15	0.70
Current Smoker, *n* (%)	20 (33.3)	22 (36.7)	χ^2^ = 0.15	0.70
Prior Myocardial Infarction, *n* (%)	10 (16.7)	12 (20.0)	χ^2^ = 0.22	0.64
Prior PCI, *n* (%)	15 (25.0)	14 (23.3)	χ^2^ = 0.05	0.83
Prior Stroke/TIA, *n* (%)	5 (8.3)	6 (10.0)	χ^2^ = 0.10	0.75
Index ACS Diagnosis: STEMI, *n* (%)	25 (41.7)	26 (43.3)	χ^2^ = 0.03	0.85
Baseline Laboratory Values				
LDL-C, mmol/L	2.8 ± 0.8	2.9 ± 0.9	t = −0.64	0.52
HDL-C, mmol/L	1.01 ± 0.30	0.98 ± 0.28	t = 0.57	0.57
Triglycerides, mmol/L^b^	1.7 (1.3–2.2)	1.8 (1.4–2.3)	Z = −0.75	0.45
Platelet count, x10⁹/L	225 ± 60	230 ± 55	t = −0.48	0.63
HbA1c, %	6.5 ± 1.0	6.6 ± 1.1	t = −0.52	0.60
eGFR, mL/min/1.73m^b^	85 ± 20	82 ± 22	t = 0.78	0.44
Medications on Admission				
Aspirin, *n* (%)	60 (100)	60 (100)	-	1.00
P2Y12 Inhibitor, *n* (%)	60 (100)	60 (100)	-	1.00
Ticagrelor	40 (66.7)	42 (70.0)	χ^2^ = 0.15	0.70
Clopidogrel	20 (33.3)	18 (30.0)	χ^2^ = 0.15	0.70
Beta-blocker, *n* (%)	54 (90.0)	52 (86.7)	χ^2^ = 0.32	0.57
ACEI/ARB, *n* (%)	48 (80.0)	50 (83.3)	χ^2^ = 0.22	0.64
Statin, *n* (%)^c^	55 (91.7)	56 (93.3)	-	1.00

a Statistic shown is the t-value for continuous variables (t-test), χ^2^ for categorical variables (chi-square test), or Z-score for non-parametric continuous variables (Mann–Whitney U test).

b Analyzed using Mann–Whitney U test due to skewed distribution.

c Analyzed using Fisher's exact test because the expected cell count for patients without statins was <5.

### 3.2 Primary endpoint analysis

**Figure 2:**
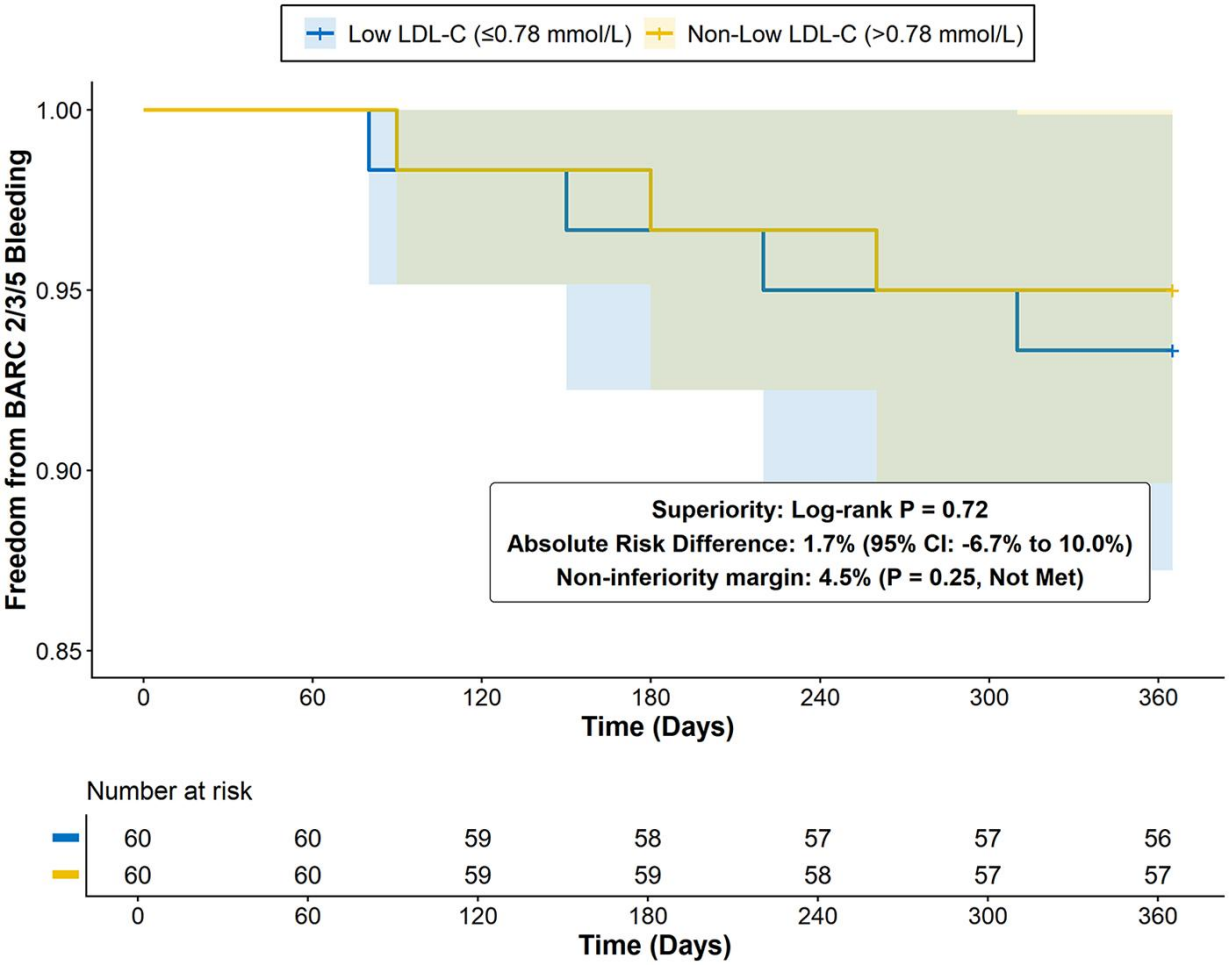
Kaplan–meier curves for the primary safety endpoint. Kaplan–Meier estimates for the cumulative incidence of the primary endpoint (BARC type 2, 3, or 5 bleeding) at 12 months. The analysis demonstrates no significant difference in event-free survival between the Low LDL-C (≤0.78 mmol/L) and Non-Low LDL-C (>0.78 mmol/L) groups (log-rank *P* = 0.72).

Over the 12-month follow-up period, the primary endpoint of clinically relevant bleeding (BARC types 2, 3, or 5) was observed in 4 patients (6.7%) in the Low LDL-C group and in 3 patients (5.0%) in the Non-Low LDL-C group, as detailed in Table 2. The absolute risk difference between groups was 1.7% (95% CI: −6.7% to 10.0%). It is important to note that with only 7 total clinically relevant bleeding events across the entire cohort, the study was structurally underpowered to generate a confidence interval narrow enough to formally assess the pre-specified non-inferiority margin of 4.5%. While the one-sided 97.5% upper confidence limit for the risk difference was 10.0%, this wide interval is an artifact of the low event rate. Consequently, this pre-specified analysis has been reframed as descriptive and exploratory rather than definitive. However, superiority testing confirmed no significant difference in bleeding risk between the two cohorts (Log-rank *P* = 0.72). These results were supported by the time-to-event analysis, where the Kaplan–Meier curves for the cumulative incidence of bleeding showed no significant divergence between the two groups during follow-up (Figure 2).

**Table 2:** Comparison of primary endpoint events at 12 months.

Endpoint	Low LDL-C Group (*n* = 60)	Non-Low LDL-C Group (*n* = 60)	Absolute Risk Difference (95% CI)	*P* value^a^
Primary Endpoint, n (%)(BARC Type 2, 3, or 5 Bleeding)	4 (6.7)	3 (5.0)	1.7% (−6.7% to 10.0%)	0.72
BARC Type 2	3 (5.0)	2 (3.3)	-	-
BARC Type 3	1 (1.7)	1 (1.7)	-	-
BARC Type 5	0 (0.0)	0 (0.0)	-	-

Data are presented as *n* (%).

a For superiority, calculated using the Log-rank test. The one-sided upper 97.5% confidence limit was 10.0%, which exceeded the 4.5% margin (*P* for non-inferiority=0.25).

These safety findings were highly consistent in the adjusted analyses. After multivariable adjustment for key clinical covariates including P2Y12 inhibitor type, the hazard ratio for clinically relevant bleeding in the Low LDL-C group compared to the Non-Low LDL-C group was 1.15 (95% CI: 0.25–5.28; *P* = 0.85). Similarly, the IPTW sensitivity analysis yielded a consistent neutral result (HR 1.20, 95% CI: 0.28–5.15; *P* = 0.80), confirming the absence of a significant superiority difference.

### 3.3 Key secondary endpoint: coagulability and platelet reactivity changes

Serial assessments of platelet and coagulation function were performed throughout the study. Analysis revealed that the achievement of Low LDL-C (≤0.78 mmol/L) was not associated with significant alterations in global coagulability or specific platelet reactivity across multiple testing modalities over the 12-month follow-up.

**Figure 3:**
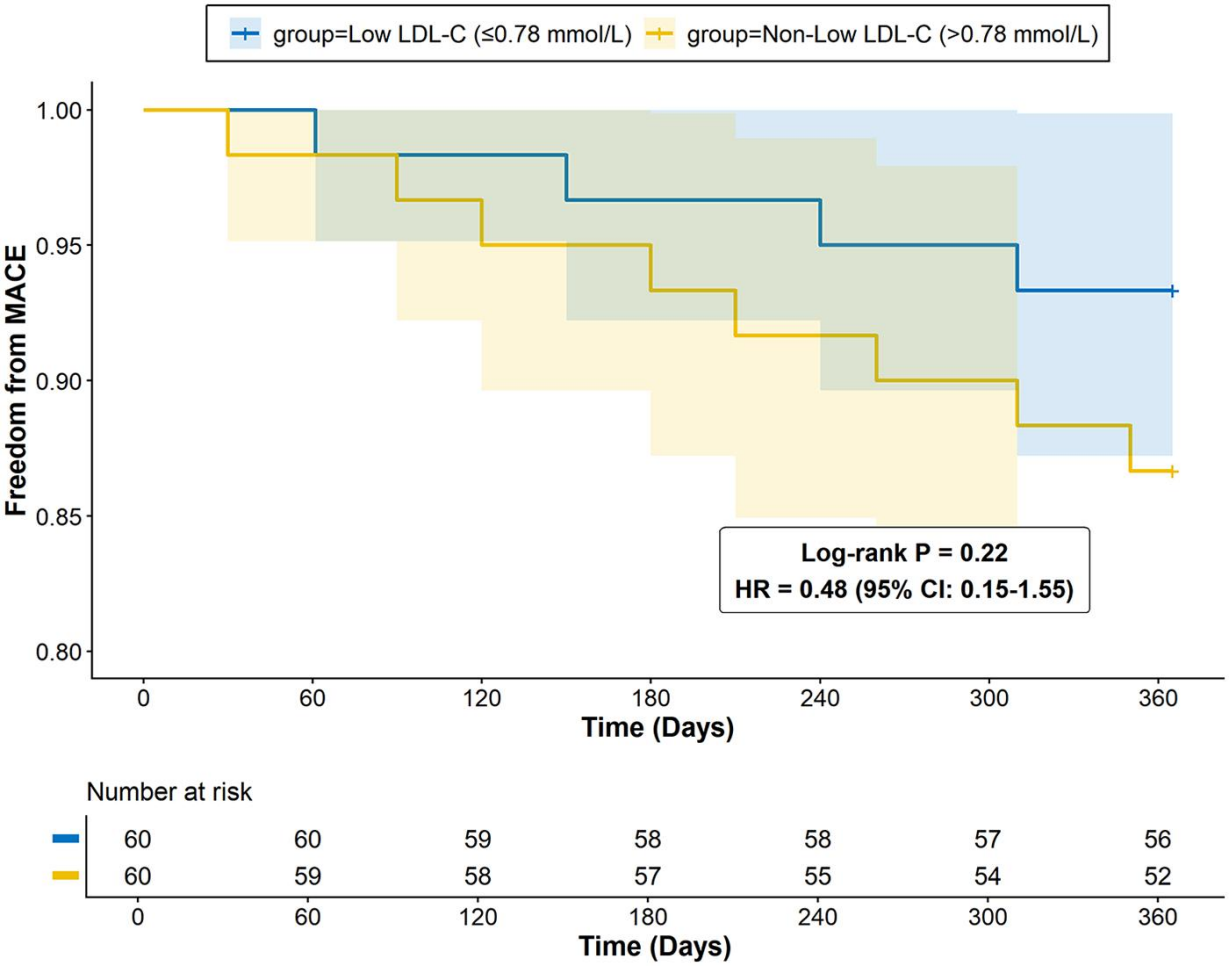
Kaplan–meier curves for the key secondary composite endpoint. Kaplan–Meier estimates for freedom from Major Adverse Cardiovascular Events (MACE) at 12 months. The curves demonstrate an early and persistent, though non-statistically significant, numerical trend favoring the Low LDL-C (≤0.78 mmol/L) group compared to the Non-Low LDL-C (>0.78 mmol/L) group (log-rank *P* = 0.22).

TEG, the prespecified primary coagulation assay, demonstrated stable and parallel trajectories in both groups. As illustrated by the representative parameter MA in Figure 3, values remained within the normal physiological range throughout the study. Baseline TEG and other platelet function parameters were well-balanced (all *P* > 0.05). A repeated-measures mixed-effects model found no significant group-by-time interaction effect for any measured parameter (all interaction *P* > 0.70). Consistently, pairwise between-group comparisons at each time point showed no statistically significant differences (all *P* > 0.05, Table 3).

**Table 3:** Comprehensive longitudinal assessment of platelet and coagulation function.

Assay (Unit)	Group	Baseline	1 Month	3 Months	6 Months	12 Months	P for Interaction^a^
TEG: R-time (min)	Low LDL-C	6.2 ± 1.1	6.1 ± 1.3	6.3 ± 1.2	6.2 ± 1.1	6.4 ± 1.3	0.92
	Non-Low LDL-C	6.3 ± 1.2	6.2 ± 1.0	6.1 ± 1.3	6.3 ± 1.2	6.2 ± 1.1	
	*P* value^b^	0.64	0.64	0.38	0.64	0.36	
TEG: K-time (min)	Low LDL-C	1.5 ± 0.4	1.6 ± 0.5	1.5 ± 0.4	1.6 ± 0.5	1.5 ± 0.5	0.88
	Non-Low LDL-C	1.5 ± 0.5	1.5 ± 0.4	1.6 ± 0.5	1.5 ± 0.4	1.6 ± 0.4	
	*P* value^b^	1	0.23	0.23	0.23	0.23	
TEG: MA (mm)	Low LDL-C	64.1 ± 5.5	63.8 ± 5.2	63.5 ± 5.8	63.9 ± 5.4	63.5 ± 6.0	0.81
	Non-Low LDL-C	63.8 ± 5.2	64.0 ± 5.0	64.2 ± 5.5	63.7 ± 5.3	64.4 ± 5.7	
	*P* value^b^	0.76	0.83	0.5	0.84	0.4	
TEG: Angle (°)	Low LDL-C	65.2 ± 6.1	64.9 ± 5.8	64.5 ± 6.3	65.0 ± 6.0	64.8 ± 6.5	0.77
	Non-Low LDL-C	65.5 ± 5.9	65.8 ± 5.5	65.9 ± 5.8	65.1 ± 6.1	65.3 ± 6.2	
	*P* value^b^	0.79	0.38	0.21	0.93	0.67	
TEG: G (kdynes/cm^2^)	Low LDL-C	9.0 ± 1.4	8.9 ± 1.3	8.8 ± 1.5	8.9 ± 1.3	8.8 ± 1.6	0.84
	Non-Low LDL-C	8.9 ± 1.3	9.0 ± 1.2	9.1 ± 1.4	8.9 ± 1.3	9.2 ± 1.5	
	*P* value^b^	0.69	0.66	0.26	1	0.16	
Platelet Count (x10⁹/L)	Low LDL-C	225 ± 60	220 ± 55	218 ± 58	222 ± 53	218 ± 52	0.88
	Non-Low LDL-C	230 ± 55	228 ± 52	225 ± 60	226 ± 57	224 ± 58	
	*P* value^b^	0.64	0.41	0.52	0.69	0.55	
CRT (%)	Low LDL-C	59.2 ± 6.8	58.8 ± 7.0	58.5 ± 7.5	59.0 ± 6.9	58.5 ± 7.2	0.95
	Non-Low LDL-C	58.8 ± 6.5	58.5 ± 6.8	58.0 ± 7.2	58.2 ± 7.0	57.8 ± 6.9	
	*P* value^b^	0.74	0.81	0.71	0.53	0.59	
ACT (s)	Low LDL-C	122 ± 14	124 ± 16	123 ± 15	125 ± 14	125 ± 15	0.76
	Non-Low LDL-C	124 ± 16	126 ± 15	127 ± 17	128 ± 16	128 ± 17	
	*P* value^b^	0.47	0.48	0.17	0.28	0.31	
RPFA (PRU)^c^	Low LDL-C	195 ± 45	190 ± 43	188 ± 44	186 ± 41	188 ± 42	0.82
	Non-Low LDL-C	190 ± 40	185 ± 42	183 ± 39	184 ± 40	182 ± 38	
	*P* value^b^	0.52	0.52	0.51	0.79	0.41	

ACT, activated clotting time; CRT, clot retraction test; G, shear elastic modulus; LDL-C, low-density lipoprotein cholesterol; MA, maximum amplitude; RPFA, rapid platelet function assay; PRU, P2Y₁₂ reaction units; TEG, thromboelastography. Data presentation: Continuous data are presented as mean ± standard deviation.

a *P* value for the group-by-time interaction effect from the repeated-measures mixed-effects model.

b *P* value for between-group comparison at each specific time point (independent samples t-test).

c RPFA was performed using the VerifyNow® P2Y₁₂ assay.

### 3.4 Key secondary endpoint: composite endpoint events

A total of 12 MACE were recorded during the 12-month follow-up. The incidence showed a numerical trend toward fewer events in the Low LDL-C group, with 4 events (6.7%), compared to 8 events (13.3%) in the Non-Low LDL-C group (Table 4). This difference did not reach statistical significance. The time-to-event analysis, using a Cox proportional hazards model, produced a hazard ratio (HR) of 0.48 (95% confidence interval: 0.15–1.55; *P* = 0.22). Corresponding Kaplan–Meier curves demonstrated an early and persistent, though non-significant, separation favoring the Low LDL-C group over the follow-up duration (Figure 3).

**Table 4:** Comparison of key secondary composite endpoint events at 12 months.

Endpoint	Low LDL-C Group (*n* = 60)	Non-Low LDL-C Group (*n* = 60)	Hazard Ratio (95% CI)	*P* value^a^
MACE, n (%)	4 (6.7)	8 (13.3)	0.48 (0.15 to 1.55)	0.22
All-cause mortality	1 (1.7)	2 (3.3)	-	-
Non-fatal myocardial infarction	2 (3.3)	5 (8.3)	-	-
Non-fatal stroke	1 (1.7)	1 (1.7)	-	-

a Calculated using the log-rank test.

### 3.5 LDL-C levels during follow-up

As designed, a significant and sustained separation in LDL-C levels was achieved between the two groups throughout the 12-month study (Table 5). Following the 2-week run-in period and treatment stabilization, the LDL-C level at the time of group assignment (study baseline) was 0.68 ± 0.14 mmol/L in the Low LDL-C group and 1.45 ± 0.35 mmol/L in the Non-Low LDL-C group (*P* < 0.001).

**Table 5:** Longitudinal LDL-C levels during follow-up.

Group	Baseline*	1 Month	3 Months	6 Months	12 Months	P for Interaction^a^
Low LDL-C						
Mean ± SD (mmol/L)	0.68 ± 0.14	0.65 ± 0.15	0.70 ± 0.16	0.69 ± 0.15	0.70 ± 0.13	0.45
Non-Low LDL-C						
Mean ± SD (mmol/L)	1.45 ± 0.35	1.41 ± 0.38	1.48 ± 0.40	1.43 ± 0.36	1.46 ± 0.39	
Between-group *P* value^b^	<0.001	<0.001	<0.001	<0.001	<0.001	

Baseline refers to the post-run-in assessment used for definitive group stratification.

a *P* value for the group-by-time interaction effect from repeated-measures analysis of variance.

b *P* value for between-group comparison at each time point. Calculated using Welch's *t*-test instead of Student's t-test, as the artificially constrained inclusion threshold (≤0.78 mmol/L) for the Low LDL-C group resulted in expected unequal variances between the two cohorts.

* The post washout evaluation used for final grouping.

**Figure 4:**
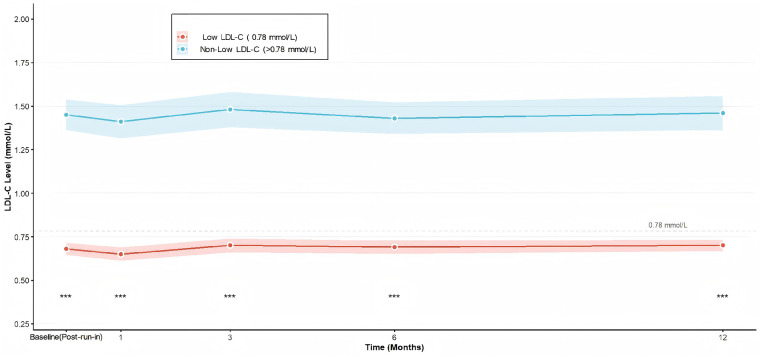
Trajectories of achieved LDL-C levels during follow-up. Mean LDL-C levels with 95% confidence intervals are plotted over time. The figure demonstrates the early, significant, and sustained separation between the Low LDL-C (≤0.78 mmol/L) and Non-Low LDL-C (>0.78 mmol/L) groups, which was maintained throughout the study. Statistical significance at each time point is denoted (***P < 0.001).

This difference remained statistically significant at all subsequent follow-up assessments (all between-group *P* < 0.001 at months 1, 3, 6, and 12). As visualized in Figure 4, LDL-C levels remained stable within each group over time, with the Low LDL-C group consistently maintaining mean levels well below the 0.78 mmol/L threshold. Repeated-measures analysis confirmed no significant group-by-time interaction (*P* = 0.45), indicating parallel and stable trajectories in both groups and verifying the robustness of the longitudinal group stratification.

### 3.6 Other secondary and safety analyses

Analysis of other prespecified secondary and safety endpoints further corroborated a similar safety profile between the two groups. As detailed in Table 6, no statistically significant differences were observed in the incidence of any severe bleeding events, including BARC type 3 or 5 major bleeding (1.7% vs. 1.7%, *P* > 0.99), TIMI major or minor bleeding (1.7% vs. 1.7%, *P* > 0.99), or other major bleeding definitions. Similarly, for exploratory ischemic endpoints, although the event rates were numerically lower in the Low LDL-C group for most outcomes, no statistically significant differences were found between the groups in the incidence of cardiovascular death, myocardial infarction, ischemic stroke, or definite/probable stent thrombosis (all *P* > 0.05).

**Table 6:** Comparison of other secondary and safety endpoints at 12 months.

Endpoint	Low LDL-C Group (*n* = 60)	Non-Low LDL-C Group (*n* = 60)	*P* value^a^
Safety Endpoints (Bleeding Events)			
BARC type 3 or 5 bleeding	1 (1.7)	1 (1.7)	1.00
TIMI major or minor bleeding	1 (1.7)	1 (1.7)	1.00
GUSTO moderate, severe, or life-threatening bleeding	1 (1.7)	1 (1.7)	1.00
ISTH major bleeding	1 (1.7)	1 (1.7)	1.00
Other Secondary Endpoints (Ischemic Events)			
Cardiovascular death	1 (1.7)	2 (3.3)	1.00
Myocardial infarction	1 (1.7)	3 (5.0)	0.62
Ischemic stroke	1 (1.7)	1 (1.7)	1.00
Definite/probable stent thrombosis	0 (0.0)	1 (1.7)	1.00

Data are presented as n (%).

a *P* values were calculated using Fisher's exact test due to the low number of expected events (expected cell counts < 5).

### 3.7 Subgroup and correlation analyses

**Figure 5:**
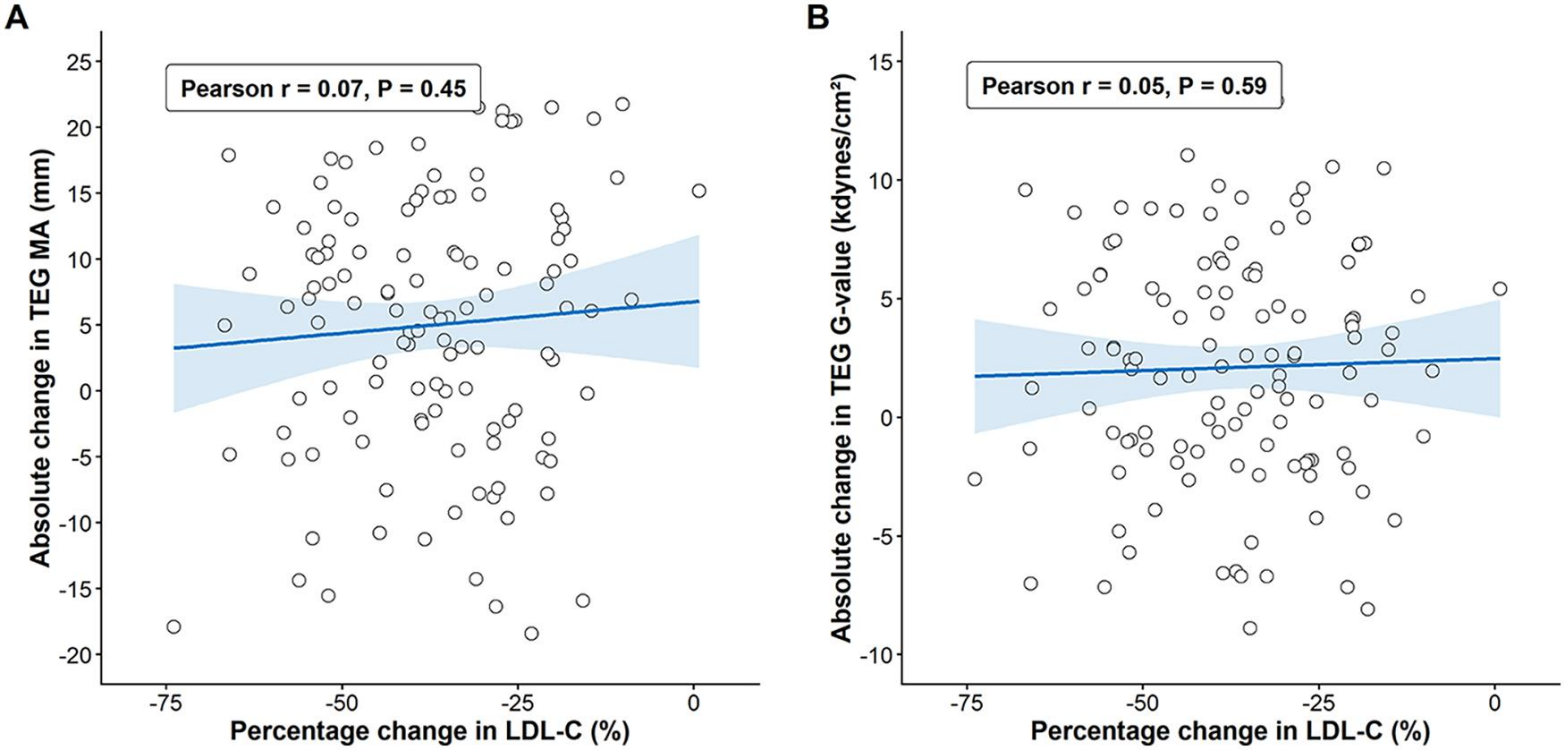
Correlation between the magnitude of LDL-C reduction and changes in thromboelastography parameters. **(A)** Scatter plot illustrating the relationship between the percentage change in LDL-C and the absolute change in Maximum Amplitude (MA) from baseline to 12 months. **(B)** Scatter plot illustrating the relationship between the percentage change in LDL-C and the absolute change in the G-value from baseline to 12 months. For both panels, each point represents an individual patient (*n* = 120). The blue line represents the linear regression fit, with the shaded area indicating the 95% confidence interval. There was no significant correlation observed in either analysis, indicating that the extent of LDL-C lowering did not influence overall clot strength.

To further explore the potential relationship between the magnitude of LDL-C reduction and coagulability or platelet reactivity, a correlation analysis was performed. As depicted in Figure 5A, there was no significant correlation between the percentage change in LDL-C from baseline to 12 months and the corresponding absolute change in the TEG MA value across the entire study cohort (Pearson r = 0.07, *P* = 0.45). This result was further corroborated by a sensitivity analysis using the TEG G-value, a derivative parameter of MA that directly quantifies clot shear strength, which also showed no significant correlation with the percent reduction in LDL-C (Pearson r = 0.05, *P* = 0.59; Figure 5B). This consistent lack of association suggests that the extent of LDL-C lowering did not influence the overall clot strength.

Furthermore, an analysis was conducted to determine if baseline hemostatic parameters could predict subsequent bleeding events. Patients were stratified into two groups based on the occurrence of any BARC type ≥ 2 bleeding during the one-year follow-up. As shown in Table 7, a comprehensive comparison of baseline platelet and coagulation function parameters revealed no significant differences between patients who experienced a bleeding event (*n* = 7) and those who did not (*n* = 113). Baseline TEG parameters (R-time, K-time, MA, Angle, G-value), platelet count, clot retraction, activated clotting time, and P2Y12 reactivity were all comparable between the two groups (all *P* > 0.05). These results indicate that neither global coagulability nor specific platelet reactivity, as measured at baseline, were associated with the subsequent risk of bleeding in this cohort.

**Table 7:** Comparison of baseline platelet and coagulation parameters between patients with and without bleeding events.

Baseline Parameter (Unit)	Bleeding Event Group (*n* = 7)	No Bleeding Event Group (*n* = 113)	*P* value^a^
TEG: R-time (min)	6.3 (5.5–7.2)	6.1 (5.4–6.9)	0.68
TEG: K-time (min)	1.5 (1.2–1.9)	1.4 (1.2–1.8)	0.75
TEG: MA (mm)	63.8 (59.0–68.0)	64.2 (60.5–67.5)	0.81
TEG: Angle (°)	65.0 (60.0–69.0)	65.5 (61.0–69.5)	0.79
TEG: G (kdynes/cm^2^)	8.7 (7.5–10.0)	8.9 (8.0–10.0)	0.72
Platelet Count (x10⁹/L)	210 (175–255)	225 (190–265)	0.55
Clot Retraction Test (CRT) (%)	58.0 (52.0–63.0)	59.5 (54.0–64.0)	0.58
Activated Clotting Time (ACT) (s)	125 (115–140)	123 (114–135)	0.61
RPFA (PRU)^b^	182 (145–215)	185 (160–215)	0.65

ACT, activated clotting time; CRT, clot retraction test; G, shear elastic modulus; MA, maximum amplitude; PRU, P2Y₁₂ reaction units; RPFA, rapid platelet function assay; TEG, thromboelastography. DData are presented as median (interquartile range) due to the highly skewed sample size distribution between groups. The Bleeding Event Group includes patients with any BARC type ≥ 2 bleeding.

a Calculated using the Mann–Whitney U test.

b RPFA was performed using the VerifyNow® P2Y₁₂ assay.

## 4 Discussion

This study investigated the association between achieved LDL-C categories following PCSK9 inhibitor initiation and global coagulability, specific platelet reactivity, and clinical outcomes in patients with ACS on DAPT. The principal finding of our study is that achieving a low LDL-C level (≤0.78 mmol/L) was not associated with an increased absolute risk of clinically relevant bleeding events compared to remaining at a moderately low LDL-C level (>0.78 mmol/L). Given the sparse bleeding events, the pre-specified non-inferiority testing was structurally underpowered; therefore, the primary safety outcome was assessed descriptively. This exploratory time-to-event analysis confirmed a comparable and flat event-free survival trajectory without any safety signal. This observation provides important clinical reassurance for intensive lipid-lowering strategies in a high-risk population. Furthermore, a comprehensive and longitudinal assessment using multiple assays, including TEG and rapid platelet function testing, revealed no significant alterations in global coagulability or specific platelet reactivity associated with the low LDL-C level over a 12-month follow-up period.

The safety of achieving exceptionally low LDL-C levels has been a subject of ongoing discussion, particularly regarding a potential increase in hemorrhagic risk. Some observational studies have suggested an association between low LDL-C concentrations and an increased risk of bleeding, especially intracerebral hemorrhage (16). However, our findings align with the robust safety data from large-scale randomized controlled trials of PCSK9 inhibitors, such as FOURIER and ODYSSEY OUTCOMES, which demonstrated no significant increase in hemorrhagic stroke or other major bleeding events despite achieving median LDL-C levels well below 1.0 mmol/L (10, 11, 29). Our study extends these findings by prospectively evaluating bleeding events using the standardized BARC definition in an ACS population, confirming that achieving intensive LDL-C reduction is not associated with compromised hemostatic safety, even in the setting of potent antiplatelet therapy. The observed neutral association with bleeding is a critical finding, suggesting that the benefits of profound lipid-lowering are not offset by an increased bleeding hazard in this specific clinical context.

A unique contribution of our study is the detailed, prospective investigation of both global coagulation function and specific platelet reactivity. Preclinical and mechanistic studies have proposed that PCSK9 may directly modulate platelet reactivity by binding to platelet surface receptors like CD36, thereby enhancing activation and thrombosis (12, 30). This raised a theoretical concern that the profound degree of LDL-C reduction achieved by these agents might alter hemostatic responses. However, our comprehensive analysis—utilizing TEG as an overall coagulation assay alongside targeted rapid platelet function testing—demonstrates no discernible impact on global coagulability or specific platelet reactivity. This finding is concordant with a recent abstract analysis from the EVOPACS trial, which also found no effect of early evolocumab initiation on ADP-induced platelet aggregation in ACS patients (31). It is plausible that any subtle pleiotropic effects of PCSK9 on hemostasis are clinically insignificant or completely overshadowed by the potent and dominant effects of guideline-directed DAPT (22). The lack of any significant correlation (Pearson r < 0.10) between the magnitude of LDL-C reduction and changes in coagulation parameters in our analysis further supports the conclusion that achieving low LDL-C is unlikely to be associated with a clinically relevant alteration in overall coagulation function. It is noteworthy that the mean P2Y12 reaction unit (PRU) values across our cohort ranged from approximately 185 to 195 PRU. This suggests that a meaningful proportion of patients, predominantly those receiving clopidogrel, exhibited high on-treatment platelet reactivity (HTPR, typically defined as PRU > 208). While we identified these high responders during our analysis, the overall incidence of ischemic events was too low to establish any statistically significant correlation between HTPR and subsequent MACE in this specific underpowered context.

While our study lacked the statistical power to definitively assess efficacy, the observed 48% relative risk reduction for MACE in the low LDL-C group, although not statistically significant, is noteworthy. This trend aligns with the foundational principle established by the IMPROVE-IT trial that “lower is better” for cardiovascular outcomes (23), and is consistent with the benefits seen when targeting the ultra-low median LDL-C levels achieved in the FOURIER trial (10). The favorable trend in MACE is likely associated with the profound and sustained reduction of LDL-C, which is known to promote the stabilization and regression of vulnerable atherosclerotic plaques. Numerous intravascular imaging studies using OCT and IVUS have provided mechanistic support for this, demonstrating that PCSK9 inhibitor therapy leads to a significant increase in fibrous cap thickness, a reduction in the lipid core, and overall plaque volume regression (32–34). These favorable morphological changes reduce plaque vulnerability and the propensity for rupture, which likely underlies the reduction in ischemic events observed in large outcomes trials and the trend seen in our cohort. Our findings, therefore, add to the body of evidence supporting the early and aggressive use of PCSK9 inhibitors to maximize plaque stabilization and improve clinical outcomes following an ACS event (35).

The clinical implications of our findings are direct and relevant. This study provides evidence to support the implementation of current guideline recommendations, which advocate for aggressive LDL-C lowering in patients with ACS to a target of <1.4 mmol/L or even <1.0 mmol/L for very high-risk individuals (15, 18, 21). Our data offer reassurance to clinicians that pursuing these ambitious targets with PCSK9 inhibitors in ACS patients receiving DAPT is safe, without being associated with an increased risk of bleeding or adverse alterations in coagulability and platelet reactivity. This supports the concept of initiating intensive lipid-lowering therapy early after an ACS event to derive the maximum clinical benefit.

Although our primary cohort consists of ACS patients without active malignancies, the hemostatic safety profile of PCSK9 inhibitors established here holds substantial translational relevance for the emerging field of cardio-oncology. Cancer survivors face an accelerated risk of atherosclerotic cardiovascular disease secondary to cardiotoxic oncological treatments and inflammatory overlaps, necessitating highly aggressive lipid-lowering strategies (36). Furthermore, malignancies and their treatments inherently disrupt the hemostatic balance, making bleeding risk a paramount and competing concern when deploying advanced cardiovascular antithrombotic or lipid-lowering agents (37). Therefore, confirming that profound LDL-C reduction via PCSK9 inhibition does not further compromise global coagulation function or increase bleeding risk in a high-risk cardiovascular population provides critical foundational evidence. This safety assurance is a necessary prerequisite for confidently extending intensive PCSK9-targeted therapies to complex cardio-oncology patients, who urgently require safe and potent cardiovascular risk mitigation without compounding their hemorrhagic vulnerability (38, 39).

This study has several strengths, including its prospective design, the focus on a well-defined and clinically important patient population, and the use of multiple, comprehensive assays to evaluate global coagulability and specific platelet reactivity over a long-term follow-up. However, we must also acknowledge its limitations. First, as a single-center, open-label study, it may be subject to inherent biases. Second, the group allocation was based on the achieved LDL-C level rather than randomization. Crucially, because recruitment for the Non-Low LDL-C group was deliberately extended to capture patients who failed to achieve the target despite PCSK9 inhibitor therapy, this comparator group is likely enriched with individuals who differ systematically from true responders. These differences may encompass unmeasured confounding factors such as variations in long-term medication adherence, distinct pharmacogenomic profiles, adverse metabolic phenotypes, and heavier underlying comorbidity burdens. Third, the extremely low incidence of bleeding events (sparse events) resulted in a wide confidence interval, rendering the study structurally underpowered for the pre-specified non-inferiority test. Consequently, our primary safety findings must be interpreted as exploratory and descriptive rather than definitive, despite the lack of any clinical safety signal. Similarly, the small sample size rendered the study underpowered to detect a statistically significant difference in the efficacy endpoint of MACE. Therefore, the observed numerical trend in MACE reduction should be considered strictly hypothesis-generating. Although we implemented multivariable adjustment and IPTW sensitivity analyses, these statistical models can only address measured covariates and cannot fully correct for the fundamental unmeasured confounding arising directly from the sampling design itself. Therefore, we explicitly state that this study does not support causal inference, and all estimations must be interpreted with strict clinical caution.

## 5 Conclusion

This exploratory analysis descriptively demonstrates that achieving an LDL-C level ≤0.78 mmol/L following PCSK9 inhibitor initiation in ACS patients on DAPT is not associated with an increased risk of significant bleeding or adverse alterations in laboratory measures of global coagulability and specific platelet reactivity. The observational data descriptively support the safety of attaining aggressive LDL-C targets in these high-risk individuals, aiding the adoption of guideline-directed therapy for better prognostic outcomes. However, given the potential for unmeasured confounding inherent in our sampling design, these findings do not support causal inference. Subsequent large-scale randomized trials are needed to definitively establish causal efficacy and safety benefits in this patient group.

## Data Availability

The original contributions presented in the study are included in the article/Supplementary Material, further inquiries can be directed to the corresponding author.
